# Epithelial Integrins Coordinate Cellular Crosstalk Through the Regulation of Cytokines During Tissue Remodeling

**DOI:** 10.3390/ijms27083497

**Published:** 2026-04-14

**Authors:** Jacob Snyder, Sanjana Dhulipalla, Whitney M. Longmate

**Affiliations:** 1 Department of Molecular and Cellular Physiology, Albany Medical College, Albany, NY 12208, USA; snyderj4@amc.edu (J.S.); dhulips@amc.edu (S.D.); 2 Department of Surgery, Albany Medical College, Albany, NY 12208, USA

**Keywords:** integrins, cytokines, secretome, crosstalk, tumor microenvironment, extracellular matrix, wound healing, fibrosis

## Abstract

Integrins are obligate αβ heterodimeric receptors that mediate cell–extracellular matrix interactions and exhibit bidirectional signal transduction across the plasma membrane. This integrin-mediated signal transduction regulates the expression of genes, a subset of which encode cytokines—small, secreted proteins that exhibit cell–cell communication in an autocrine or paracrine manner to regulate cell survival, proliferation, migration, ECM remodeling, and the immune response. This review examines epithelial integrins in the regulation of paracrine-acting cytokines that crosstalk to immune and stromal cells to coordinate normal and pathological tissue remodeling. Contexts explored include wound repair, fibrosis, and cancer.

## 1. Introduction

The introductory subsections below will introduce integrins (Section 1.1), cytokines (Section 1.2), and the regulation of cytokines by integrins (Section 1.3) ahead of exploring this relationship in context (Section 2 and Section 3).

### 1.1. Integrins: Cell Adhesion and Beyond

**Figure 1:**
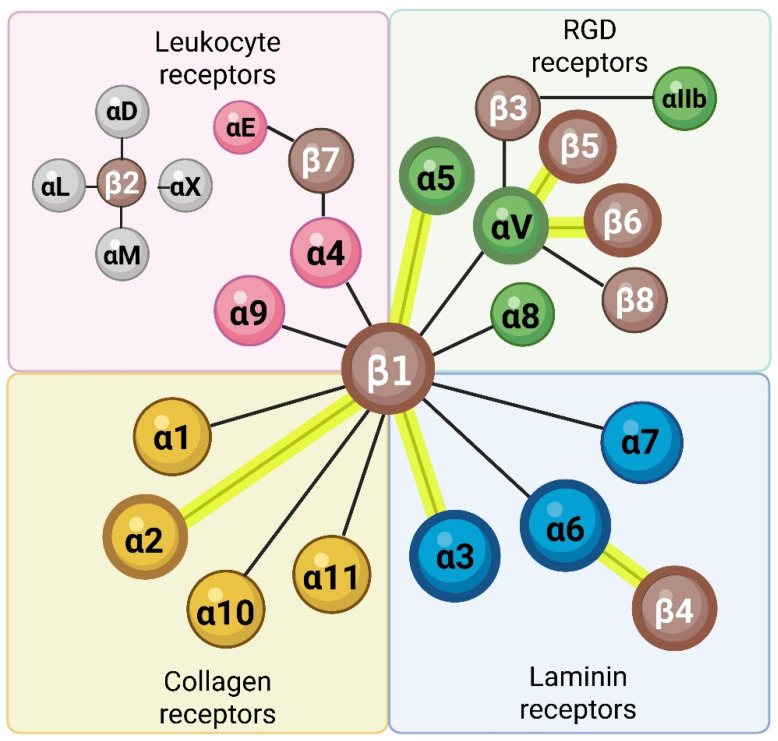
The integrin family of cell adhesion receptors and their ligand-binding specificities. A classical categorization is shown and is mainly based on ligand-binding specificity. Integrins are expressed as transmembrane receptors on the cell membrane and bind ECM proteins with extracellular domains. Eighteen α subunits can dimerize with eight β subunits in limited combinations to form twenty-four distinct integrins with different ligand-binding specificities. Major epithelial integrins are highlighted and include laminin receptors, α6β4 and α3β1, collagen receptor, α2β1, and RGD-binding receptors α5β1, αvβ5, and αvβ6. While traditionally known as adhesion receptors, the roles of integrins are vast and varied, and in certain contexts include mediating intercellular crosstalk via the regulation of secreted factors. This figure was adapted from earlier work [1]. Created in BioRender. Longmate, W. (2026) https://BioRender.com/lc476k4 (accessed on 1 April 2026).

Integrins are the major cell surface receptors that mediate adhesion to the extracellular matrix (ECM) [1]. Integrins are obligate heterodimers consisting of an α and a β subunit, each having a single-pass transmembrane domain, a cytoplasmic domain that interacts with cytoskeletal proteins, and a large extracellular domain that binds to specific ECM ligands. Eighteen α subunit monomers can dimerize with eight β subunits in limited combinations, yielding a set of twenty-four integrins with distinct yet often overlapping ligand-binding specificities [1]. Integrins are often classified by their ligand-binding capability (Figure 1). Some integrins, called RGD-binding integrins, can recognize the tripeptide RGD (Arg, Gly, Asp) sequence present in certain ligands including fibronectin, vitronectin, and tenascin [1]. Collagen and laminin, however, lack accessible RGD sequences. Major epithelial cell integrins include laminin receptors, α6β4 and α3β1, collagen receptor, α2β1, and RGD-binding integrins, α5β1, αvβ5, and αvβ6 (Figure 1; highlighted) [2].

Structurally, integrins on the cell membrane can take on an “active” or “inactive” conformation and often shift between states [1]. Integrins bind to ECM ligands via their extracellular domains and simultaneously interact with cytoskeletal proteins via their cytoplasmic domains [1]. In the “active” conformation, integrins more readily bind their ECM ligands. These interactions form a physical linkage of the ECM to the cytoskeleton, which is essential for regulating cell shape, adhesion, polarization, and motility [1,3,4,5,6]. Integrins are also known to interact directly and indirectly with a variety of signaling effectors. This function as a signaling conduit allows integrins to act as bidirectional signal transducers [1,3,7,8], capable of “outside–in” signaling (e.g., an extracellular cue such as ECM binding promotes intracellular pathway modulation) and “inside–out” signaling (e.g., a cytoplasmic interaction promotes modulation of the activation state of an integrin to alter its affinity for extracellular ligands) [1,7,9]. This integrin-mediated signal transduction regulates many cell functions that are critical for normal as well as pathological processes, including survival, migration, proliferation, ECM remodeling, and gene expression [1,4,7]. For detailed information on integrin structure and signaling, please refer to the following comprehensive and seminal reviews [1,10].

While all integrins share some similar features, they differ functionally. Integrin α6β4 is a key transmembrane protein found in hemidesmosomes, which are multi-protein structures that connect epithelial cells to the underlying basement membrane. The primary role of integrin α6β4 is structural, supporting the integrity of these adhesion structures, while it has minimal involvement in signal transduction [11]. Integrin αVβ6 is a major transforming growth factor-β (TGFβ) activator; it can directly bind to the latency associated peptide (LAP) to activate TGFβ via mechanotransduction [12]. We will discuss TGFβ signaling in more detail below (Section 1.4), and in the context of fibrosis (Section 3.1) and cancer (Section 3.2). Integrins α3β1 (a laminin receptor), α2β1 (a collagen receptor), α5β1 (a fibronectin receptor), and αvβ5 (a vitronectin receptor) commonly utilize focal adhesion kinase (FAK), among other pathways (discussed further in Section 1.4), to transduce signals governing cell adhesion, survival, and migration [13,14,15,16,17].

**Figure 2:**
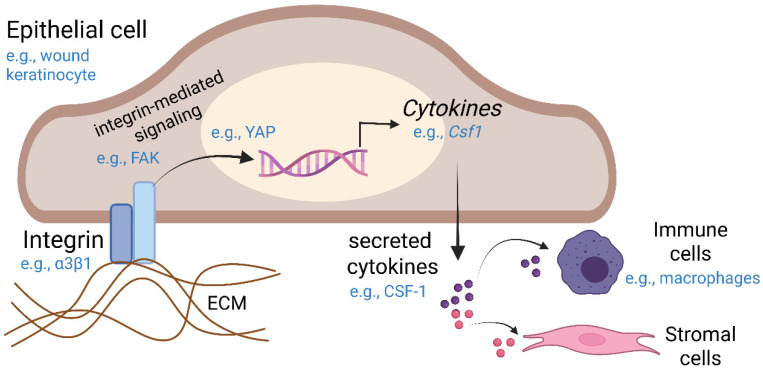
Epithelial integrins regulate cytokines to support distinct cells in the milieu. This generic model depicts an ECM-bound epithelial integrin promoting cytokine transcription via an integrin-mediated signaling pathway. The resulting cytokines, once secreted, act as chemical messengers that support distinct cells in the microenvironment, including the immune, stromal cells, and vascular compartments. This type of intercellular crosstalk is thought to be especially critical during tissue remodeling processes, such as wound repair, and during pathological remodeling events, such as tumorigenesis/cancer progression and fibrosis. For example, during wound healing, integrin α3β1 on wound keratinocytes signals through the FAK/YAP axis to induce the expression of CSF-1, supporting the macrophage population [19], as described in the main text. Created in BioRender. Longmate, W. (2026) https://BioRender.com/o437bo5 (accessed on 1 April 2026).

Within tissues, integrins are expressed on the surface of epithelial, stromal, and immune cells and are well known to regulate autonomous cellular functions in each cell type. This review will examine emerging roles for integrins on epithelial cells in the regulation of secreted factors into the microenvironment (i.e., the secretome). In particular, the focus will be on integrin-dependent, epithelial-derived cytokines of the secretome that support distinct cells within the milieu (Figure 2), primarily in the contexts of wound healing, fibrosis, and tumor progression. Other components of the epithelial integrin-regulated secretome (apart from cytokines) including exosomes, growth factors, proteases, and matrix proteins, as reviewed in [18], may also be briefly mentioned.

### 1.2. Cytokines: Chemical Messengers That Regulate the Microenvironment

Cytokines are a broad category of small, secreted proteins and signaling molecules released by cells to regulate an array of cellular processes within tissue, including inflammation. As chemical messengers, cytokines often bind to target cell receptors and control cell growth, differentiation, and tissue repair, as reviewed [20]. Cytokines have distinct and overlapping functions and affect nearly every biological process, including aging, repair, disease pathogenesis, and the immune response. Classic classes of cytokines include interleukins (ILs), interferons (IFNs), colony-stimulating factors (CSFs), tumor necrosis factor (TNF), and chemokines. Mesenchymal growth factors such as TGF, connective tissue growth factor (CTGF; also known as cellular communication network factor 2 (CCN2)), and vascular endothelial growth factor (VEGF) are also considered multifunctional cytokines. General descriptions of the cytokine classes are provided below, and relevant cytokines will be discussed further in the following sections (Section 2 and Section 3).

In general, ILs act as key communicators to and amongst immune cells to coordinate broader immune responses and regulate inflammation [21]. IFNs can be produced by host cells in response to pathogens to coordinate antiviral defenses, and they are known for their tumor-suppressive roles [22]. CSFs are known to support the growth, differentiation, and/or recruitment of macrophages (CSF-1 and -2) and/or granulocytes (CSF-2 and -3) [23]. TNF is a central cytokine in inflammatory reactions—regulating inflammatory gene expression—and is capable of inducing apoptotic, pyroptotic, or necroptotic cell death [24]. Interestingly, TNF can also mediate cell survival primarily by activating nuclear factor-κB (NF-κB) signaling to promote the expression of pro-survival genes [24]. Chemokines act as chemoattractants to guide migration of immune cells to sites of injury, inflammation, or infection [20]. VEGF cytokines act as potent stimulators of angiogenesis and lymphangiogenesis and can regulate vascular permeability [25]. TGFs (TGFα and TGFβ) regulate essential cellular processes such as proliferation and differentiation, and can control aspects of repair, ECM formation, and the immune response [26]. TGFβ signaling, canonically through Smad proteins, plays a central role in immune and tissue regulation. Notably, the ability of TGFβ to promote the differentiation of quiescent fibroblasts into active, contractile, ECM-producing myofibroblasts is a key contributor to tissue fibrosis [27], a topic discussed in detail below (Section 3.1). Furthermore, CTGF/CCN2 is a key mediator of TGFβ signaling and is also considered a profibrotic cytokine that is involved in tissue remodeling, fibrosis, and inflammation [28]. TGFβ signaling, and other integrin-mediated signaling pathways, are discussed in more details below in Section 1.4.

### 1.3. Regulation of Cytokines by Integrins

While first described as cell adhesion receptors, the role of integrins has expanded to include a variety of functions, both cell-autonomous and paracrine. Many ‘paracrine’ signaling functions of epithelial integrins are thought to occur through the regulation of secreted factors, including cytokines, which can modulate crosstalk to distinct cell types within the stroma, as discussed below. Mechanistically, the regulation of secreted factors by epithelial integrins is thought to occur primarily at the level of gene regulation (Figure 2) [29], although this regulation can also occur at the level of secretion or via integrin-mediated cell surface recruitment, as reviewed in [30]. Furthermore, endocytosed integrins can serve as active intracellular signaling platforms rather than being degraded or recycled, as reviewed in [31], and in such a way may regulate secreted factors. Moreover, epithelial integrins can mediate exocytosis or cargo within exosomes. Indeed, integrin-dependent signaling has been shown to coordinate exocytic machinery during neurite sprouting [32], although it remains to be seen whether this is a general mechanism of secretome regulation by integrins in other contexts. Interestingly, integrins themselves are often contained in exosome cargo, and exosome-derived integrins have been linked to cancer progression, as reviewed in [33].

### 1.4. Integrin-Dependent Signaling

While integrin-dependent signaling is wide-ranging, we will introduce below the major integrin signaling pathways that are implicated in the regulation of cytokines, including integrin-mediated mechanotransduction, FAK/Src, yes-associated protein (YAP)/transcriptional enhanced associate domain (TEAD), and TGFβ/Smad pathways.

FAK and Src family kinases are among the most widely studied effectors of integrin signaling [13,14,15,16,17]. The FAK-Src signaling axis can be activated by integrin-mediated adhesion in epithelial cells [34,35,36,37], and as such, acts as a critical link between integrins and mechanotransduction, serving as a key converter of physical forces into biochemical signals. Integrins, acting as mechanical sensors on the cell surface, bind to the ECM and, upon experiencing tension or structural changes, recruit FAK, which subsequently initiates intracellular signaling cascades that regulate transcriptional programs and cell behavior [38].

Upon integrin-mediated adhesion, FAK phosphorylates itself on Y397, generating a high-affinity binding site for the SH2 domain of Src. FAK-bound Src can then phosphorylate additional residues on FAK (e.g., Y861, Y925), thereby generating binding sites for other adaptor/signaling proteins. In this way, FAK serves as an “activatable scaffold” that links integrin–ECM adhesions to downstream signaling effectors such as the Rac1 GTPase or the mitogen-activated protein kinases (MAPKs), extracellular signal-regulated kinase-2 (ERK-2), c-Jun N-terminal kinase (JNK), or p38 [7,16,39]. Furthermore, FAK can mediate YAP activation by promoting YAP entry into the nucleus where interaction with the TEAD-family of transcription factors allows for the transcription of YAP target genes. Key cytokines regulated by YAP include CCL2, IL-33, IL-25, IFNγ and CSF-1 [19,40,41].

Integrins, particularly αVβ6 and αVβ8, regulate the cytokine TGFβ primarily by binding to the LAP of inactive TGFβ and contracting to dissociate LAP, which liberates active TGFβ from the ECM [12]. Active TGFβ can then bind TGFβ cellular receptors which canonically activate Smads intracellularly to form a Smad complex that can enter the nucleus and act as a transcription factor that regulates transcriptional programs controlling mainly cell growth, proliferation, and differentiation [12]. TGFβ can also activate alternative non-Smad signaling pathways, including MAPK/ERK, PI3K/AKT, and JNK/p38 [42]. Notably, establishment of TGFβ feed-forward loops are known to sustain the differentiation of fibroblasts into active myofibroblasts, which are hypercontractile and produce excessive ECMs, perpetuating fibrotic phenotypes [27].

The following sections will highlight examples of epithelial integrin-regulated cytokines in mediating supportive crosstalk to stromal and immune cells during tissue repair (Section 2), and in the pathological contexts of fibrosis and cancer (Section 3).

## 2. Integrin Regulation of Cytokines in Wound Healing

The regulation of growth factor, cytokine, and chemokine bioavailability represents a key checkpoint in governing the inflammatory phase of wound repair, as dysregulated or persistent inflammation is a major contributor to the development of chronic nonhealing wounds and fibrotic disease. In addition, coordinated cytokine signaling is essential for directing protease-driven degradation of fibrillar collagen and other ECM proteins necessary for effective tissue remodeling during later stages of wound healing [43].

Despite the epithelium being spatially separated from the connective tissue, extensive communication is established through diffusion of epithelial-derived growth factors and cytokines to various other cellular compartments throughout the tissue microenvironment. In this context, there are certain epithelial integrins that stimulate distal wound cells such as fibroblasts, endothelial cells, and inflammatory cells through production of growth factors and cytokines [44,45,46,47]. This mechanism thereby facilitates epithelial integrin-dependent paracrine signaling, which is the focus of the current review and will be explored in the context of cutaneous wound healing (below) and in the pathological contexts of fibrosis and cancer (Section 3).

It is becoming increasingly clear that the epithelial integrin-mediated secretome serves to support stromal cell function during wound repair. Several roles for epithelial integrins in mediating paracrine crosstalk with other cell types in the wound microenvironment have been identified, mainly in the context of cutaneous wound healing, as discussed below.

Epidermal integrin α3β1, a laminin binding integrin, plays a central role in regulating paracrine crosstalk between keratinocytes and multiple stromal and immune cell populations during wound repair. Keratinocyte α3β1 regulates the wound fibroblast compartment by inducing IL-1α expression, which in turn activates cyclooxygenase-2 (Cox-2)/prostaglandin E2 (PGE2) signaling to restrain TGFβ-driven myofibroblast differentiation [48]. Zheng et al. utilized 5- and 10-day wounds from epidermal α3-knockout mice as their primary experimental model while also supplementing with in vitro studies using immortalized mouse keratinocytes that express and lack α3β1. In vitro manipulations included treatment with IL-1α neutralizing antibodies, recombinant IL-1RA (receptor antagonist), and IL-1 receptor-targeting small interfering RNA (siRNA) to demonstrate the functional role of IL-1α signaling in mediating α3β1’s effects on fibroblasts. This study showed that α3β1-expressing keratincytes secrete more IL-1α and less IL-1RA compared to α3-null cells, creating a pro-inflammatory microenvironment. This pathway may engage a regulatory feedback loop, as fibroblast-derived PGE2 has been shown to suppress TGFβ-mediated myofibroblast differentiation in an autocrine manner [49]. Of note, IL-1α mediated stromal activation has also been observed in tumor settings, where tumor-associated IL-1α stimulates tumor-associated fibroblasts in pancreatic cancer models, supporting the conceptual parallel between tumor-associated fibroblasts and wound myofibroblasts [50,51] (see Section 3.2 for expanded discussion in the cancer context).

Moreover, a study from our group identified a role for keratinocyte α3β1 in supporting the wound macrophage population through FAK-YAP/TEAD-dependent induction of macrophage colony-stimulating factor, CSF-1 (Figure 2), with 3- day wounds from α3 epidermal knockout (KO) mice exhibiting reduced epidermal CSF-1 expression and diminished macrophage recruitment [19]. We utilized an inducible, epidermis-specific α3 knockout mouse model generated using a tamoxifen-induced Cre-lox system to excise the *Itga3* gene, resulting in deletion of integrin α3β1 from keratinocytes of adult mice to bypass potentially confounding compensatory mechanisms. Consistent with a broader role for integrins in immune regulation, inhibition of epithelial α3β1 integrin signaling reduces the expression of immune cell-homing interleukins IL-6 and IL-8 along with macrophage chemoattractant MCP-1 [52], whereas epidermal β1 integrin overexpression enhances IL-1α secretion [52]. In this study, colonic Caco-2 epithelial cells and lung A549 cells were treated with an anti-α3 integrin cross-linking antibody prior to stimulation with pro-inflammatory cytokines. Anti-α3 antibody treatment suppressed IL-1-stimulated secretion and mRNA expression of IL-6, IL-8, and MCP-1. Similar suppression was observed for TNF-α-stimulated IL-6 secretion.

Interestingly, cutaneous wound angiogenesis is also tightly regulated by integrin-dependent paracrine signaling originating from epidermal keratinocytes, although roles for cytokines in this context are less clear. Briefly, keratinocyte integrin α3β1 promotes wound angiogenesis through keratinocyte secretion of the pro-angiogenic factor, MRP-3 [53], which is not itself considered a cytokine. Another keratinocyte integrin, α9β1, functions later in wound healing to act as a break on α3β1-mediated vascular growth in order to facilitate regression/remodeling of excessive neovasculature [54]. Furthermore, enhanced wound vascularization observed in α2-null mice supports a suppressive role for integrin α2β1 in angiogenic regulation [55]. Together, these studies highlight the coordination of vascular remodeling mediated by epidermal integrins.

Furthermore, given the dynamic nature of wound healing, it is likely that integrin–cytokine regulation is highly stage-dependent, especially since distinct phases are characterized by shifting cellular behaviors and signaling requirements. During the early inflammatory phase, cytokines such as IL-1 and IL-6 are rapidly induced [56,57] and may rely on integrin signaling to coordinate immune cell recruitment, adhesion, and activation. In contrast, during later stages of tissue repair and remodeling, cytokines such as TGFβ predominate [58] and are more closely linked to integrin-dependent regulation of fibroblast activation, extracellular matrix deposition, and tissue contraction. These temporal differences suggest that integrin signaling is not static, but dynamically modulates cytokine networks in a phase-specific manner. More integrin–cytokine studies that fully encompass the wound healing timeline are needed to elucidate the dynamic nature of this regulation.

## 3. Integrin Regulation of Cytokines in Pathological Tissue Remodeling Contexts

As in tissue repair (Section 2), the epithelial integrin-mediated regulation of cytokines is prevalent in pathological tissue remodeling and is more well-studied in these contexts. Below, we will explore this regulation across multiple organ systems in the contexts of fibrosis (Section 3.1) and cancer (Section 3.2). In particular, TGFβ signaling is heavily implicated as an integrin-mediated cytokine with important roles in these pathological contexts [59]. For intstance, epithelial integrin-mediated signaling can promote TGFβ expression or activation which can crosstalk to distinct cells in the milieu, such as fibroblasts, and can influence disease progression.

### 3.1. Fibrosis

Fibrosis is the pathological overgrowth, hardening, and/or scarring of tissue as a result of chronic inflammatory processes, leading to organ dysfunction and failure. It is driven by sustained cytokine signaling that promotes fibroblast activation, ECM deposition and remodeling, and aberrant cell crosstalk, as reviewed in [60,61]. Fibrosis affects many organs, most commonly the lungs, heart, liver, and kidneys, and is a leading cause of morbidity and death worldwide [61,62]. Fibrosis was long seen as a progressive and irreversible disease but it is now viewed as a dynamic process with the potential for therapeutic interventions [61,62,63].

Epithelial integrins have emerged as critical regulators of fibrotic progression through their ability to mediate cytokine activation and downstream signaling. The most heavily implicated pro-fibrotic cytokines are the TGFβ superfamily and their downstream effectors, Smad proteins, and CTGF/CCN2 [64,65]. Three structurally similar isoforms of TGFβ, TGFβ1-3, are encoded by three different genes in mammals and are expressed in a tissue-specific manner under control of a unique promoter [64,66]. The most prevalent isoform in fibrosis is TGFβ1 [64,67].

Various epithelial integrins have been implicated as upstream regulators of TGFβ signaling in fibrosis in different tissues. Much focus of current research is on identifying therapeutic targets to mitigate fibrotic disease progression. Epithelial integrins present as excellent candidates, owing to their accessibility on the cell surface and well-defined signaling dogmas. Perhaps unsurprisingly, there are instances whereby integrin signaling has been demonstrated to have an opposite anti-fibrotic effect, consistent with known complexities in integrin biology described in other contexts such as cancer (Section 3.2). However, several studies have explored both molecular and pharmacodynamic targeting of epithelial integrins or their signaling effectors to reduce pro-fibrotic phenotypes. Highlighted below are select recent findings concerning epithelial integrins regulating fibrotic phenotypes via TGFβ signaling and therapeutic approaches that have targeted this signaling paradigm.

#### 3.1.1. Cardiac Fibrosis

One study showed that epithelial integrin subunit αv-mediated mechanoparacrine TGFβ1 signaling promoted fibrous scar formation predominantly in immature fibrous areas after myocardial infarction in mice [68]. Epithelial integrin α2β1 was demonstrated to coordinate stiffness-controlled release of the pro-inflammatory cytokine IL-6 by cardiac fibroblasts cultured on polyacrylamide gels with varying elasticities grafted to collagen, contributing to a chronic inflammatory state driving a fibrotic milieu [69]. Furthermore, there are non-epithelial examples of integrin-related cytokines that drive fibrosis in cardiac models. Experiments using a transverse aortic constriction mouse model and in vitro culture of neonatal rat fibroblasts and cardiomyoctes revealed the secretory protein integrin beta-like 1 (ITGBL1), a β-integrin-related ECM protein, as a key mediator of fibroblast-cardiomyocyte crosstalk during cardiac remodeling in heart failure, partially through TGFβ-Smad2/3 signaling [70]. Lentiviral short hairpin RNA (shRNA) knockdown of the calcium-activated chloride channel ANO1 was shown to significantly reduce TGFβ- and IL-6-induced adhesion and migration of cardiac fibroblasts by inhibiting integrin and FAK expression [71]. Tamsulosin, an α1 adrenergic blocker widely reported to have cardioprotective effects, is a drug with therapeutic potential in preventing myocardial infarction through regulation of the integrin-linked kinase (ILK)-TGFβ-Smad signaling pathway [72].

#### 3.1.2. Liver Fibrosis

In patients with metabolic dysfunction-associated steatohepatitis and hepatocellular carcinoma, it was found that levels of galectin 3-binding protein (LGALS3BP) were positively correlated with TGFβ1, LGALS3BP directly bound to epithelial integrin αv, and depletion of LGALS3BP slowed hepatic fibrosis by limiting the availability of TGFβ1 [73]. Furthermore, a truncated version of milk fat globule-epidermal growth factor 8, previously shown to have an anti-fibrotic role in liver disease, attenuated liver fibrosis in animal models by inhibiting integrin–TGFβ receptor interactions [74].

#### 3.1.3. Kidney Fibrosis

In a chronic kidney disease model, collagen I and TGFβ1 synergistically increased connexin-43 hemichannel activity and ATP release upon integrin α2β1 binding in renal tubular epithelial cells [75]. Interestingly, in preclinical models of chronic kidney disease, treatment with the αvβ8-targeting monoclonal antibody MEDI8367 ameliorated renal fibrosis [76]. Conversely, epithelial β8 integrin subunit degradation and activation of TGFβ1/Smad3 signaling contributed to pericyte–myofibroblast transition and renal fibrosis in a model of diabetic kidney disease [77].

#### 3.1.4. Pulmonary Fibrosis

The chemokine CCL20, produced primarily by type 2 alveolar epithelial cells, interacts with integrin α5β1 on lung fibroblasts and enhances TGFβ/Smad signaling, promoting myofibroblast differentiation in pulmonary fibrosis [78]. The β-galactoside-binding lectin galectin-3 binds to αv integrins αvβ1, αvβ5, and αvβ6, as well as the TGFβR2 subunit and promotes TGFβ signaling, contributing to pulmonary fibrogenesis [79]. Functional studies of *Itga5*, the gene encoding epithelial α5 integrin subunit, showed that upon pharmacodynamic treatment with 13-methylpalmatine, an active compound from the Chinese goldthread plant (*Coptis chinensis*), *Itga5* expression was downregulated and downstream activation of TGFβ/Smad signaling was inhibited, ameliorating bleomycin-induced pulmonary fibrosis [80].

Taken together, these findings suggest a critical role for epithelial integrins in regulating fibrosis via control of cytokine signaling dynamics. These findings also suggest therapeutic promise to treat fibrosis, with epithelial integrins and their signaling effectors as key targets. Of course, integrin targeting is not without its challenges, and preclinical studies do not always predict clinical success, as discussed in more detail in Section 4 below.

### 3.2. Cancer

Epithelial integrins are also well-established regulators of various processes involved in tumorigenesis and tumor progression, such as survival, tumor initiation, angiogenesis, migration, invasion, and metastasis [81,82,83]. Indeed, abnormal expression and functionality of epithelial integrins has been implicated in many cancer types for several decades now. Similar to fibrosis, regulation of cytokine signaling, particularly TGFβ, by epithelial integrins has been identified as a key contributor to maintenance of the tumor microenvironment (TME) [82,84]. The TME is a complex and dynamic region surrounding tumors, chiefly comprising immune cells, cancer-associated fibroblasts, endothelial cells, and pericytes, that supports tumor survival, growth, and metastasis. The region is hypoxic due to the high proliferation rate of the cancer cells and abnormal blood vessel structure, which has been shown to contribute to immune tolerance and drug resistance [85]. TME characteristics are often dependent on primary tumor site, tumor stage, and individual patient differences [86]. In addition to hypoxia, signaling by TGFβ and other cytokines is known to promote epithelial-to-mesenchymal transition (EMT), whereby cancer cells acquire invasive and stem-like properties, as well as increased ECM stiffness that facilitates cancer cell migration, invasion, and metastasis [87].

Several epithelial integrins, particularly αv, β1, and β3-containing integrins, have been shown to be important modulators of cytokine signaling in cancer, some with pro-tumor and some with anti-tumor effects dependent on cancer cell type, integrin subtype, ligand binding, and TME conditions [81]. TGFβ also has a notable temporal-dependent tumorigenic role, generally having anti-tumor effects in earlier cancer stages, and, conversely, pro-tumor effects in later cancer stages [87]. Generally, it is thought that TGFβ acts as a tumor suppressor by inhibiting epithelial cell proliferation, inducing cell cycle arrest, and promoting apoptosis, but as the tumor and TME evolves, TGFβ shifts toward promoting tumor progression in later stages by driving EMT, enhancing cell migration and invasion, stimulating ECM remodeling, and fostering an immunosuppressive microenvironment [87,88].

As with fibrosis, the major focus of current research is developing therapeutic approaches to delay, stop, and reverse cancer progression. The association between epithelial integrins and downstream cytokine signaling across different cancers makes them intriguing therapeutic targets. There has been some preclinical evidence that integrin antagonists, particularly targeting β3 integrins, could be effective in halting cancer progression [84,89,90,91,92,93,94], though Phase II and III clinical trials with the RGD-binding integrin antagonist, Cilengitide, disappointingly failed to meet primary endpoints, which has hindered development of other integrin antagonist drugs [95,96]. Development of successful widespread treatment has also been hindered by integrin expression and function being highly tumor type- and disease state-dependent [81]. These topics are discussed further in Section 4.2 below.

Several studies have explored the mechanisms by which epithelial integrins exert control over cytokine signaling dynamics, how this control goes awry in cancer cells, and how to potentially target it. Reviewed below are recent findings involving epithelial integrin–cytokine (dys)function contributing to solid tumor initiation, growth, and metastasis in prevalent cancer types, as well as therapeutic approaches that have been attempted.

#### 3.2.1. Breast Cancer

Many epithelial β1 integrins, in particular integrin α3β1, have long been established as required for mammary tumorigenesis [34,81,97,98]; though, conversely, it has been demonstrated that integrin α2β1 can act as a metastasis suppressor in transgenic mice [99], and α3β1 itself may have anti-tumorigenic effects in other cancer contexts, such as prostate cancer, as demonstrated in an orthotopic model [100]. Similarly, integrin αvβ6, known to stimulate TGFβ signaling, exhibits both pro- and anti-tumor effects, dependent on cancer stage and TGFβ activation state in xenograft mouse models [101,102]. Many of these differing oncogenic effects may be explained by compensatory up- and downregulation of other epithelial integrins with overlapping functions but that have divergent roles in regulating TGFβ signaling. It has more recently been reported that expression of enhancer of zeste homolog 2 (EZH2) promotes osteolytic metastasis of breast cancer via regulation of TGFβ signaling in triple-negative breast cancer in vitro and in vivo, which in turn activates β1 integrins and FAK [103]. Intercellular adhesion molecule 1 (ICAM1) was found to interact with epithelial integrins to activate EMT through TGFβ/Smad signaling, promoting a bone metastatic phenotype, also in a triple-negative breast cancer model [104]. Again, in triple-negative breast cancer, connexin-43 was found to mediate metastatic state via an integrin αv-TGFβ-Smad3 signaling axis [105]. The adapter protein kindlin-2, which binds to the cytoplasmic domain of epithelial integrins, was identified to have a role in stabilizing β1 integrin-TGFβ1 receptor complexes and regulating their downstream signaling, both in vitro and in preclinical in vivo mouse models of triple-negative breast cancer [106]. The small-molecule inhibitor KBU2046 inhibited TGFβ1 and effectively suppressed cell motility in triple-negative breast cancer cells [107], indicating that abrogating TGFβ1 signaling may be a therapeutic avenue for the treatment of aggressive breast cancer.

#### 3.2.2. Lung Cancer

Epithelial integrin α5β1, a fibronectin receptor, has been heavily implicated in non-small-cell lung cancer (NSCLC), the most common type of lung cancer, comprising approximately 85% of all cases [108,109,110]. In one study, integrin α5β1, in coordination with insulin-like growth factor binding protein 2 (IGFBP2), was found to increase resistance to the epidermal growth factor receptor (EGFR) inhibitor drug Gefitinib by activating a STAT3-CXCL1 signaling axis both in vitro and in an NSCLC tumor xenograft model [111]. Other epithelial integrins have also been shown to contribute to integrin–cytokine dynamics in lung cancer. In lung adenocarcinoma, epithelial integrin αvβ8 has recently been demonstrated to regulate the chemokine CCL5, which promotes macrophage infiltration and polarization in tumor xenograft and tail vein metastasis models [112]. In lung cancer patients with EGFR mutations, there is evidence of crosstalk between cancer cells and pericytes that regulate tyrosine kinase inhibitor sensitivity through IL-32-β5 integrin signaling [113]. The tyrosine kinase inhibitor, Nintedanib, has been conjugated to integrin αvβ6 ligands, which reduced TGFβ-induced EMT in a model of NSCLC [114]. Additionally, an integrin αvβ1-PYK2-STAT3-VGF nerve growth factor signaling axis was also shown to promote NSCLC and be responsive to PYK2 and STAT3 inhibitors in a tumor xenograft model [115].

#### 3.2.3. Colorectal Cancer

Epithelial integrin-mediated TGFβ signaling has also been implicated in colorectal cancer. For example, integrin β5-TGFβ signaling promotes malignancy [116], and a periostin–TGFβ feed-forward loop stimulates crosstalk between tumor and stromal cells in liver metastases of colorectal cancer [117]. Furthermore, integrin–chemokine signaling has been particularly well studied in colorectal cancer. Metastasis to the liver has been shown to be promoted by integrin αvβ6-SDF-1-CXCR4 signaling in a syngeneic metastasis model [118], and αv integrins have similarly been found to contribute to peritoneal metastasis of colorectal cancer through regulation of CXCL2-CXCR2 signaling [119]. Treatment with the microRNA miR-145-5p effectively inhibited CXCL1 signaling and integrin α2, suppressing proliferation and migration of colorectal cancer cells [120]. Epithelial integrin αvβ3 also triggers EMT, promoting colorectal cancer malignancy via HIF-1α-STAT3 regulation of secretory phosphoprotein-1 (SPP1) secreted by tumor-associated macrophages in a tumor xenograft model [121].

#### 3.2.4. Melanoma/Skin Cancer

Epithelial integrin–cytokine dynamics remain underexplored in melanoma and other skin cancers; however, a few recent studies show that αvβ3 and αvβ5 in ectosomes contribute to crosstalk to endothelial cells during angiogenesis. Specifically, αvβ5-VEGF signaling and αvβ3-TNFα signaling are involved, with the αvβ5-VEGF axis playing a larger role in angiogenic promotion [122]. In addition, stimulation of toll-like receptor 3 (TLR3) in a melanoma mouse model, whose target genes encode α4, α5, and β1 integrin subunits, as well as TGFβ and IL-10, enhanced migration of adipose-derived mesenchymal stem cells as part of a stress response pathway [123]. Furthermore, integrin α3β1 in hair bulge stem cells was shown to promote skin tumorigenesis via CTGF/CCN2 expression in a conditional knockout mouse model of skin cancer [124].

#### 3.2.5. Other Cancers

Additionally, epithelial integrin–cytokine dynamics have been explored in other cancer types including pancreatic cancer [125,126,127,128], liver cancer [129,130,131], thyroid cancer [132], and ovarian cancer [133,134]. To highlight an innovative therapeutic approach, a “stapled” α-helix named 5a on the neuroendocrine secretory protein chromogranin-A allowed for homing to αvβ6 and/or αvβ8-positive tumors of the pancreas and prostate, respectively. This suggested that the modified peptide has potential as a ligand, acting as a vehicle to deliver anti-cancer agents or as an inhibitor of TGFβ signaling downstream of αvβ6 and αvβ8 [135,136].

Epithelial integrin regulation of cytokine signaling has been accelerating as a research focus in recent years, with links established across many different cancer types, but often with a small pool of key molecules, namely TGFβ, ILs, and chemokines. Therapeutic advancement continues to be a challenge, especially due to the heterogenous nature of cancerous tumors and their frequent development of resistance to treatment, as well as systemic cytotoxic effects of reagents. Intriguingly, in a very recent study, a unique modification to the practice of using lipid nanoparticles to deliver RNA interference (RNAi) oligonucleotides to tumors, termed self-agglomerating nanohydrogels (SANGs), was introduced and demonstrated to exhibit accumulation and retention in both primary and metastatic tumor models of ovarian, breast, and colorectal cancer in mice and rats. SANGs have the potential to mitigate the challenges that accompany RNAi oligonucleotide delivery to tumors, namely their rapid clearance, nuclease susceptibility, and plasma membrane impermeability [137]. Therapeutic targeting of integrins in the cancer context is also a complex challenge, given the inconsistent roles of integrins across cancer types and cancer stages. Nevertheless, epithelial integrins and their signaling effectors remain strong candidates for therapeutic targets in treating cancers, either by targeting them directly, or by exploiting them for integrin-targeted drug delivery, as discussed further in Section 4.2 below. Of course, their utility would be further enhanced with more complete understanding of specific integrin functions and their intricate relationship with the milieu.

### 3.3. Integrin–Cytokine Signaling in Fibrosis and Cancer: A Comparative Study

As discussed above, integrin–cytokine signaling is a central regulatory axis in both fibrosis and cancer, and while highly context-dependent, there are some general comparisons that can be drawn. In fibrosis, integrin–cytokine signaling is relatively focused, primarily involving a fibroblast-centric, repair-driven, TGFβ feed-forward loop whereby stiffness reinforces fibroblast activation with a relatively linear progression.

In cancer, the same signaling components are repurposed into a highly adaptive, multi-node network. While fibrosis is fibroblast-centric, as mentioned above, cancer involves a multi-compartment TME. Commonly in cancer, stiffness supports invasion, EMT, and niche formation, while cytokine-driven immunologic changes support immune evasion. However, cancer-related integrin–cytokine signaling is complex, adaptive, highly context-dependent, and can shift between tumor-suppressive and pro-tumorigenic, perhaps mirroring the stage-dependent shift in TGFβ tumorigenic function described above. Moving forward, there is a need to more thoroughly understand these intricacies towards effective therapeutic targeting (discussed further in Section 4.2).

## 4. Conclusions

### 4.1. Integrins as Regulators of Repair and Disease

Integrins have emerged as central regulators of tissue homeostasis and disease, not only through their canonical roles in adhesion and mechanotransduction, but also through their capacity to shape the epithelial secretome. By governing cytokine transcription, activation, release, and spatial presentation, epithelial integrins act as bidirectional signaling hubs that coordinate dynamic crosstalk between epithelial, stromal, and immune compartments. In the wound healing context, this regulation enables tightly controlled inflammation, angiogenesis, and matrix remodeling. In contrast, persistent or dysregulated integrin–cytokine signaling—most prominently involving TGFβ, ILs, chemokines, and growth factors—drives pathological remodeling in fibrosis and cancer, promoting fibroblast activation, immune modulation, tumor progression, and therapeutic resistance.

While there is a large body of evidence to support the role of epithelial integrins in cytokine-mediated tissue regulation, it is important to note that many experimental methods highlighted herein, including the use of pharmacological inhibitors and knockdown approaches, are not without limitations. These limitations include potential off-target effects, incomplete inhibition/knockdown, a lack of specificity, induced stress responses, and the development of compensatory and adaptive mechanisms. Furthermore, it is also important to note that, while this review focuses on the integrin-mediated regulation of cytokines, cytokines have also been known to regulate integrin activity through altering integrin clustering, conformational changes, and cell–matrix interactions [138], which underscores the high likelihood of integrin–cytokine feed-forward signaling loops that amplify and sustain cellular responses in both physiological and pathological contexts.

### 4.2. Integrins and Integrin-Related Molecules as Targets of Therapy: Challenges and Potential Future Applications

The possibility of therapeutically targeting epidermal integrins in the treatment of chronic wounds, fibrosis, or skin cancer is intriguing since the effect would not be limited to the epidermal compartment but would extend to integrin-regulated cytokines, impacting the microenvironment in a potentially powerful way. However, developing integrin-targeted therapeutics has proven challenging due to the complexity of the integrin family, the broad range of integrin functions, and their widespread expression across cell types. This complexity is further compounded by the fact that integrin activity is governed by a balance between active and inactive states, regulated through multiple mechanisms, including ligand binding, lateral protein interactions, conformational changes, and trafficking [139].

The therapeutic targeting of integrins has been established. Vedolizumab, for example, is an antibody targeting α4β7 that is utilized to inhibit lymphocyte trafficking to the gut mucosa for the management of ulcerative colitis and Chron’s disease [140,141]. In a recent review, it was stated that about ninety kinds of integrin-targeting therapeutics were in clinical trials as integrin antagonists, including antibodies, antibody–drug conjugates, synthetic mimetic peptides, and small molecules, for the treatment of several pathologies, including cancers, autoimmune diseases, viral diseases, and fibrotic diseases [139]. In the cancer clinic, integrin-blocking RGD mimetic, Cilengitide, was utilized with limited success and may serve as a cautionary tale [142,143]. Cilengitide was proposed to reduce tumor angiogenesis by inhibiting RGD-binding integrins on endothelial cells; however, this approach is likely confounded by the presence of RGD-binding integrins on other cell types within the microenvironment. Moving forward, successful application of integrin-targeting agents will require careful consideration of their effects on the entire milieu, both directly via integrin-expressing cells and indirectly through integrin-regulated secretory signaling.

Additionally, RGD mimetics target only RGD-binding integrins, overlooking others such as laminin-binding integrins like keratinocyte α3β1, which play important roles in wound healing and tumor biology, partly through regulation of a supportive secretome. Although this potential remains clinically unexplored, further preclinical work is needed to define these context-dependent functions. Notably, the high-affinity peptide LXY30 binds to α3β1 [144,145] and, while not directly modulating its activity, may serve as a targeted delivery tool—for example, to deliver therapeutics or inhibitory microRNAs to tumor cells with high α3β1 expression.

To date, clinical strategies targeting integrins have focused largely on inhibition; however, integrin targeting should not be limited to this approach. Since many integrins promote wound healing, enhancing their activity may benefit chronic or non-healing wounds, for example, through ECM-inspired biomaterials that deliver exogenous ligands or using stapled peptides [146]. Similarly, as some integrins exhibit anti-tumorigenic or anti-fibrotic roles in specific contexts, promoting their function may also have therapeutic applications in those instances as well.

Another approach, aside from integrin targeting, could involve direct delivery of integrin-regulated cytokines, which may be particularly applicable to the treatment of hard-to-heal wounds. Indeed, treatment of wounds with IL-2, CSF-1 or CSF-2 has been shown to accelerate wound healing in animal models [147,148,149]. Conversely, disruption of IL-1 signaling had healing benefits in a murine model [150], indicting potential for cytokine antagonists in certain contexts.

Across organ systems and tumor types, recurring integrin-dependent cytokine axes highlight conserved signaling paradigms while underscoring context specificity that complicates therapeutic targeting. Although clinical translation of integrin antagonists has faced challenges, growing mechanistic insight into integrin-mediated cytokine control offers renewed opportunities for precision interventions. A deeper understanding of how epithelial integrins integrate mechanical cues with inflammatory and growth factor signaling will be essential for developing strategies to restore tissue balance and limit chronic disease progression.

## Data Availability

No new data were created or analyzed. Data sharing is not applicable to this article.

## Abbreviations

The following abbreviations are used in this manuscript:

ECM	extracellular matrix
RGD	arginine (Arg), glycine (Gly), aspartic acid (Asp) tripeptide sequence
TGF	transforming growth factor
LAP	latency-associated peptide
FAK	focal adhesion kinase
IL	interleukins
IFN	interferons
CSF	colony-stimulating factors
TNF	tumor necrosis factor
CTGF	connective tissue growth factor
CCN2	cellular communication network factor 2 (another name for CTGF)
VEGF	vascular endothelial growth factor
NF-κB	nuclear factor-κB
YAP	yes-associated protein
TEAD	transcriptional enhanced associate domain
SH2	Src homology 2
Rac1	Ras-related C3 botulinum toxin substrate 1
GTP	guanosine triphosphate
MAPK	mitogen-activated protein kinase
ERK	extracellular signal-regulated kinase
JNK	c-Jun N-terminal kinase
CCL2	C-C motif chemokine ligand
PI3K	phosphatidylinositol 3-kinase
AKT	protein kinase B
Cox-2	cyclooxygenase-2
PGE2	prostaglandin E2
siRNA	small interfering RNA
*Itga3*	gene encoding α3 integrin subunit
MCP-1	monocyte chemoattractant protein-1
mRNA	messenger RNA
MRP-3	multidrug resistance protein-3
ITGBL1	integrin beta-like 1
shRNA	short hairpin RNA
ANO1	anoctamin-1
ILK	integrin-linked kinase
LGALS3BP	galectin 3-binding protein
ATP	adenosine triphosphate
TGFβR2	TGFβ receptor 2
*Itga5*	gene encoding α5 integrin subunit
TME	tumor microenvironment
EMT	epithelial-to-mesenchymal transition
EZH2	enhance of zeste homolog 2
ICAM1	intercellular adhesion molecule 1
NSCLC	non-small cell lung cancer
IGFBP2	insulin-like growth factor binding protein 2
EGFR	epidermal growth factor receptor
PYK2	proline-rich tyrosine kinase 2
STAT3	signal transducer and activator of transcription 3
SDF-1	stromal cell-derived factor-1 (also called CXCL12)
CXCR	C-X-C motif chemokine receptor
HIF-1α	hypoxia-inducible factor-1α
SPP1	secretory phosphoprotein-1
TLR3	toll-like receptor 3
RNAi	RNA interference
SANG	self-agglomerating nanohydrogel
